# Salivary detection of human Papilloma virus 16 & 18 in pre-malignant and malignant lesions of oral cavity: Is it feasible in Pakistani context of Socio-Cultural Taboos?

**DOI:** 10.12669/pjms.315.7093

**Published:** 2015

**Authors:** Iqbal A. Muhammad Khyani, Masood A. Qureshi, Talat Mirza, M. Umar Farooq

**Affiliations:** 1 Dr. Iqbal A. Muhammad Khyani, MBBS, DLO, FCPS, FRCS. Associate Professor, Department of E.N.T, Head & Neck Surgery, Dow University of Health Sciences, Dow Medical College and Civil Hospital, Karachi – Pakistan; 2 Prof. Masood A. Qureshi, M.Sc, PhD. Professor and Head, Department of Physiology, Dow University of Health Sciences, Dow International Medical College, Karachi – Pakistan; 3 Prof. Talat Mirza, MBBS, M.Phil, PhD. Professor and Head, Department of Pathology, Dow University of Health Sciences, Dow International Medical College, Karachi – Pakistan; 4 Prof. Dr. M. Umar Farooq, MBBS, DLO, FCPS, FICS, FRCS. Head of Department, ENT-Head & Neck Surgery, Dow University of Health Sciences, Dow Medical College and Civil Hospital, Karachi – Pakistan

**Keywords:** Pre-malignant lesions, Oral squamous cell carcinoma, Salivary diagnosis, Biomarkers, Human papilloma virus

## Abstract

**Objective:**

To evaluate salivary detection of HPV-16 & 18 would be feasible and informative biomarker for oral pre-malignant and malignant lesion in our population.

**Methods:**

This non-interventional, case control study was carried out at department of E.N.T, Head and Neck Surgery, Dow University of Health Sciences, Dow Medical College and Civil Hospital Karachi, Pakistan between July 2011 to December 2012. Total of 105 cases were recruited. These were divided in three groups ‘A’, ‘B’ & ‘C’ having 35 subjects each. Group‘A’ constitutes patients having strong clinical evidence of oral pre-malignant lesions (PML). Group ‘B’ includes histologically proven oral squamous cell carcinoma (OSCC) and Group ‘C’ comprised disease free subjects as controls. After taking informed consent, relevant clinical history was recorded on institutional approved performa. Saliva from all subjects was procured by standard ‘drooling method’. Samples were stored at +4°C and later transferred to Laboratory to store at-20°C before further process. Samples were centrifuged at 4500 rpm for 15 minutes at 4°C. Cell pellets sediments were used for identification of HPV-16 & 18 by real-time PCR method. Data was entered and analysed using SPSS version 16. P-value of 0.05 was taken as standard.

**Results:**

In group ‘A’, HPV-16 was detected in 3 (8.6%) cases while HPV-18 was not detected in any of the subject. In group ‘B’, HPV-16 was detected in 07 (20%) while HPV-18 was found in 06 (17.1%) cases. Mixed HPV-16 and HPV-18 were found in 02 (5.7%) cases. In group ‘C’, HPV-16 was detected in 03(8.6%) while HPV-18 was not detected in any of the subjects. Significant relationship was observed between the groups for HPV-18 detection (P= 0.002) while for HPV-16, no significant association was found (P= 0.245).

**Conclusion:**

HPV infection for the causation of oral cancer cannot be fully established possibly due to small sample size. More over differences in genetic makeup, environment, indulgence in peculiar risk factor habits, sexual practices and difficult evaluation of the acquisition of viral load due to socio-cultural and religious restrictions could be the reason.

## INTRODUCTION

Oral cancer is the sixth most frequent malignancy all over the globe.^1^ It is the common cancer in developing countries, representing the leading cause of death. It accounts for almost 40% of all cancers in the Indian subcontinent, and contributed one-third of the world burden of oral cancer. It is one of the major health concerns as it has a rising trend in younger population.^2^-^4^

Carcinoma of oral cavity is the second most frequent malignant tumour for both the gender in Pakistan. It constitutes 20-35% of all cancers seen in various public hospitals in Karachi and slightly less in other regions of Pakistan.^5^,^6^ Increasing numbers of cases are being consistently reported in younger age groups. Never the less it is a major killer in our population.^7^,^8^

It is believed that cumulative effects of nutritional deficiency, dietary habits, bad oral hygiene, mal-directed sharp teeth and infection with human papilloma virus (HPV) is implicated for oral carcinogenesis. Exposure to some of these risk factors varies significantly between ethnic groups and geographical location. In Pakistan, the major risk factors for oral cancer are areca nut (betel nut, chalia, supari), betel quid (paan), tobacco chewing, naswar, paan masala (ghutka, mawa) and poor nutrition. Due to religious prohibition, alcohol consumption and sexual promiscuity is not a prevalent habit in Karachi. This is in contrary to recent controversial John’s Hopkins study, claiming that cancer is a bad luck happening due to random mutation arising during DNA replication in normal, non-cancerous stem cells in about two third of the cases, not either related to heredity or ecological factors.^9^

Link between human papilloma virus (HPV) and squamous cell carcinoma of the head and neck was suggested in the past. Recently the association is more established as HPV-16 & 18 was the most detectable virus in salivary samples, serum and biopsy cell blocks of the patients with PMLs & OSCC.^10^

Diagnosis and subsequent therapeutic intervention of PMLs and OSCC is currently based on examination, histopathology and staging. Histological examination remains the gold standard for diagnosis of malignant oral lesions. Biopsy is an invasive technique with surgical implications, technique limitations for professionals and psychological implications for most patients. Recently there is a paradigm switch from histopathology to molecular methods of disease diagnosis and saliva has been used for molecular diagnostics by analysing genomics, proteomics and salivary transcriptomes. Oral fluid meets the demand for non-invasive, easily accessible bio-fluid of human body that nurtures a wide spectrum of biological analytes, informative for clinical diagnostic application.

In Pakistan, saliva has never been explored as a diagnostic medium to detect biomarkers for oral PMLs and oral cancer. We attempted to investigate whether detecting HPV-16 & 18 would be feasible to diagnose PML and OSCC in our population.

## METHODS

This non-interventional, case control study was carried out at department of E.N.T, Head and Neck Surgery, Dow University of Health Sciences, Dow Medical College and Civil Hospital Karachi, Pakistan between July 2011 to December 2012. Patients of any age, sex or ethnic group visiting out patients clinic having strong clinical evidence of PML, histologically proved, untreated OSCC at any stage and identical disease free subjects, willing to participate as controls, were included in the study. Subjects with prior history of treatment for loco-regional or distant malignancy and subjects with history of immune deficiency or autoimmune disorders were excluded.

The participants were divided in three groups ‘A’, ‘B’ & ‘C’ having 35 subjects in each. Group ‘A’ constitutes patients having strong clinical evidence of oral PML. Group ‘B’ includes histologically proven OSCC and Group ‘C’ constitutes disease free subjects as controls. After taking informed consent, relevant clinical history was recorded on institutional approved performa. Saliva from all recruits was procured as per standard ‘drooling method’. Samples were stored at +4°C and later transferred to Dow Diagnostic, Research & Reference Laboratory to be stored at -20°C before further process. Samples were centrifuged at 4500 rpm for 15 minutes at 4°C. Cell pellets were used for identification of HPV-16 & 18 by Real-time PCR method.

### PCR for identification of HPV-16 & 18 in saliva

### (a) DNA Extraction

Total DNA was extracted from all the samples using PureLink® Viral RNA/DNA Kits (Invitrogen Life Technologies, Carlsbad USA) for purification of viral nucleic acids according to the manufacturer’s protocol. Briefly; the collection tubes having saliva samples were centrifuged at 15,000 rpm. Pellets were re-suspended in 200 µl lysis buffer. Negative control was prepared by adding 100 µl of HPV-Neg control provided with amplification kit to the tube labelled C*neg*. Proteinase K 25 μl and 200 µl Buffer AL per 200 µl lysate was added. The solution was mixed immediately by pulse-vertexing for 15 seconds and incubated at 56°C for 10 minutes. The pH of the lysate must be acidic (<7.0) to obtain maximum binding of DNA to QIAamp membrane. Ethanol (96–100%) per 200 µl lysate was added and mixed again by pulse-vertexing for 15 seconds and briefly centrifuged to remove drops from inside the lid.620 µl of the lysate was applied to the QIAamp Spin Column (in a 2 ml collection tubes). This centrifuged at 8000 rpm for one minute. The QIAamp spin column was placed in a clean 2 ml collection tube and the tube was discarded containing the filtrate. The above two steps were repeated until the whole lysate was loaded. A maximum of 700 µl can be loaded onto the QIAamp spin column. The column was washed with 500 µl Wash Buffer (W5) with ethanol. Centrifuged again at 8000 rpm for one minute and flow through was discarded. The wash step was repeated with 500 µl of wash buffer once. The spin column was centrifuged at maximum speed for one minute to remove any residual wash buffer. The spin column was placed in a clean 1.7 ml recovery tubes. Eluted with 50 µl sterile RNase-free water supplied with the kit. Incubated at room temperature for one minute. The spin column was again centrifuged at maximum speed for one minute to elute nucleic acids. The spin column was discarded from the recovery tube contained purified viral nucleic acids. 12.5 µl of extracted DNA was used in the HPV genotype analysis using Real-time PCR.

### (b) Real-time PCR

Genotyping for HPV-16 and HPV-18 in isolated DNA from patient’s samples were performed using Real-time PCR machine (SmartCycler II, Cepheid, USA) using the Real-time PCR Kit HPV-16/18 Real-TM Quant (Sacace Biotechnologies, Italy).

### (i) Principle of Assay

Kit HPV 16/18 Real-TM Quant is based on multiplex Real-time amplification. Amplification results of HPV-16 DNA were detected on the FAM/Green channel, HPV-18 DNA were detected on the ROX/Orange channel and β-globin gene used as internal control was detected on the JOE/HEX/Yellow channel.

### (ii) Protocol

The required numbers of tubes were prepared (Number of tests + 3 standards and 1 negative control). PCR- buffer-FRT 20 µl of Hot Start DNA Polymerase in 15 µl of reaction mixture was added into each tube with samples and controls. 12.5 µl of extracted DNA sample was added to appropriate tube and 4 controls were prepared. 10 µl of Quantitative Standards HPV (QS1 HPV, QS2 HPV, and QS3 HPV) was added into labelled tubes.

### (iii) Real Time Amplification

The tubes were capped and transferred into Real Time Thermal Smart Cycler. The Smart Cycler (Cepheid, USA) instrument was programmed as per manual of instruction. The positions of the tubes were programmed and the concentrations of the Quantitative Standards entered in the JOE (Human DNA), FAM (HPV-16) and ROX (HPV-18) channels in order to generate standard curves.

### (iv) Data Analysis and Interpretations

**Fig.1:**
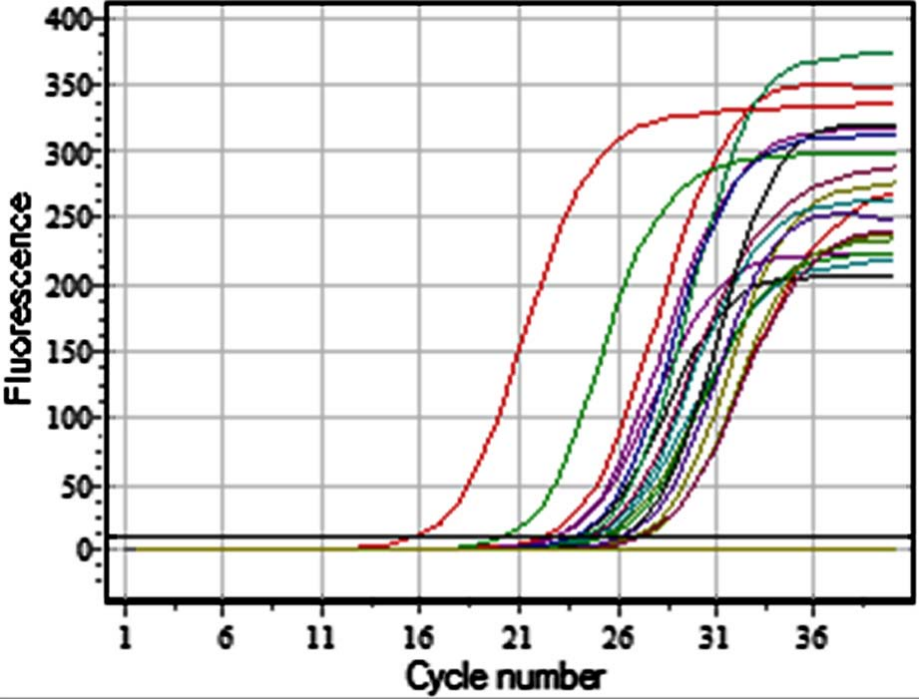
Crossing of fluorescence curves detected in one go; internal control (Human DNA) on JOE/HEX/Yellow Channel, HPV-16 on FAM/Green Channel, HPV-18 on ROX/Orange channel.

The software of Smart Cycler Real-time PCR was utilized for the interpretation of results all the way through the presence of crossing of fluorescence curve with the threshold line. As per the software, internal control (Human DNA) is detected on the JOE/HEX/Yellow channel, Human Papilloma virus type 16 (HPV 16) on the FAM/Green channel and HPV 18 on ROX/Orange channel. (Fig.1)

Data was analysed using SPSS version 16. Statistical analysis of the data was computed to see relationship among the groups by applying Pearson Chi-Square test. P-value of 0.05 was used as reference value.

## RESULT

Out of 105 subjects 70(66.7%) were males and 35(33.3%) were females with male/females ratio of 2:1. The overall minimum age of the recruited subjects was 20 years and maximum was 80 years with a mean of 43.2 years. Over all minimum age in males was 20 years and maximum was 75 years with a mean age of 43.7 years. In females over all minimum age was 21 years and maximum was 80 years with mean of 42 years. (Table-I)

**Table-I:** Gender distribution and descriptive analysis of age.

Group	No. of cases	Gender		Age in Years			
		Male	Female	Minimum	Maximum	Mean	Std. Deviation
A	35	25(71.4%)	10(28.6%)	20.00	75.00	39.3	15.9
B	35	24(68.6%)	21(60%)	26.00	80.00	46.7	14.4
C	35	21 (60%)	14 (40%)	23.00	70.00	43.5	13.7
Total	105	70 (66.7%)	35 (33.3%)	-	-	-	-

In group ‘A’, majority of the cases were having oral submucosal fibrosis (OSF). It was found in 27(77.1%) cases. Leukoplakia was found in 10(28.6%). Erythroplakia was detected in 06(17.1%) cases. All these were found alone or in combination with other pre-malignant lesions. 02(5.7%) cases were diagnosed clinically as lichen planus alone.

The subjects recruited in group ‘B’ were staged according to TNM classification of AJCC (2002-3), 02(5.7%) cases were T_1_ stage, 12(34.3%) were T_2_, 18(51.4%) were T_3_ and 03(8.6%) were T_4_ stage at presentation. 20(57.1%) cases were having no clinically palpable nodes (N_0_), 9(25.7%) were having N_1_, 05(14.3%) were having N_2_ and 01(2.9%) was having N_3_ nodal status. Distant metastasis ‘M’ was not found clinically in any of the recruited subjects (Table-II).

**Table-II:** Pre-malignant lesions Group ‘A’ (n-35)&TNM staging of OSCC Group ‘B’ (n-35).

Group	Pre-malignant Lesions							Number of Cases					
A	Oral Sub-mucosal fibrosis							27(77.1%)					
	Leukoplakia							10(28.6%)					
	Erythroplakia							06(17.1%)					
	Lichen Planus							02(5.7%)					
	Total							35 (100%)					

TNM Classification												
B	Tumour size ‘T’ (cms)					Cervical nodes ‘N’ (Clinical)					Distant Metastasis ‘M’	
	T_1_	T_2_	T_3_	T_4_	Total	N_0_	N_1_	N_2_	N_3_	Total	M_0_	M_1_
	02 (5.7%)	12 (34.3%)	18 (51.4%)	03 (8.6%)	35 (100%)	20 (57.1%)	09 (25.7%)	05 (14.3%)	01 (2.9%)	35 (100%)	35 (100%)	00 (-)

In group ‘A’, HPV-16 was detected in 3 (8.6%) cases while HPV-18 was not detected in any. In group ‘B’, HPV-16 was detected in 07 (20%) cases while HPV-18 was detected in 06 (17.1%) cases. Mixed HPV-16 and HPV-18 were found in 02 (5.7%) cases. In group ‘C’, HPV-16 was detected in 03(8.6%) while HPV-18 was not detected in any of the subject. (Table-III).

**Table-III:** High-risk human papilloma virus-16 &18 detected by Real-time PCR.

No	Genotypes	Group ‘A’	Group ‘B’	Group ‘C’	Total
1.	HPV-16	03 (8.6%)	07 (20%)	03 (8.6%)	13 (12.4%)
2.	HPV-18	Nil	06 (17.1%)	Nil	06 (5.7%)
3.	Mixed* (HPV 16 & 18)	Nil	02 (5.7%)	Nil	02 (01.9%)
Total		03 (8.6%)	15 (42.9%)	03 (8.6%)	21 (20.0%)

* Both inclusive Pearson Chi Square analysis between the groups.

P- value for HPV-16, 0.245 - Not significant P- value for HPV-18, 0.002 - Significant.

Statistical analysis was performed to compare the qualitative relationship among the groups by using Pearson Chi-Square test. The relationship of salivary HPV-16 detection was not found significant among the groups (P-0.245) while salivary HPV-18 detection was found significant (P-0.002).

## DISCUSSION

Most controversial issues relating to role of HPV in head and neck cancer, particularly OSCC are frequency of HPV, viral load acquisition through oro-genital route or otherwise and its diagnostic methodology.^10^ Its prevalence reported in OSCC vary from <5% to 100%.^11^

Modes of transmission of HPV in head and neck mucosal districts have not been fully resolved. It is proposed that multiple pathways are involved. HPV association was found to be more pronounced with the history of perverted sexual practices.^12^ Oro-genital contact, peri-natal transmission and sexual transmission specially with oral sex, multiple sex partners and possibly mouth to mouth transmission as in kissing had been implicated.^13^ It is believed that oral sex, including fellatio and cunnilingus, is the main mode of transmission of HPV infection to oral cavity.^10^ In the west, HPV is one of the most common viruses transmitted by sexual behaviours in both males and females. In many Muslim dominated countries including Pakistan, religious and socio-cultural values strictly discourage sexual perversions and promiscuity. Therefore protection from sexually transmitted HPV infection is taken for granted.

In Pakistan, HPV screening for both OSCC and cervical cancer is not commonly practiced due to socio-cultural reasons that prohibit asking enquiries related to risk factors for acquiring HPV load like oral sex and multiple partners. Therefore the data relating to acquisition of HPV infection is grossly deficient.^14^,^15^ The gravity of the situation is further aggravated due to stigmas attached to privacy of sexual practices. Many of the patients are either shy of expressing true situation or even retaliate due to socio-cultural and religious taboos that prohibit investigation of all matters pertaining to sex and sexually transmitted diseases. This is the major barrier for epidemiological surreys related to HPV incidence and prevalence in Pakistan. As the sexual non-promiscuity in our society could well be a myth, so the knot is still tied relating to association of HPV in OSCC. In the present study, we faced the same barrier. Consequently, enquires related to risk factors for acquisition of viral load were not included in data collection performa.

Etiological role of HPV in head and neck carcinogenesis was first proposed by Syrjanen et al in 1983.^16^ Subsequently, several studies have supported this proposal on the basis of well proved epitheliotropic nature of HPV, morphological similarities between oral and pharyngeal mucosa with genital epithelium and strongly established role of high risk HPV in cervical cancer.^17^,^18^

In a study from Quetta, the capital city of Balochistan province of Pakistan reported detection rate of HPV DNA in 24.5% (47/200) in a group of Pakistani subject visiting the dental department having normal oral cavity. They used oral tissue scraping for DNA detection by Real-time PCR method. Out of 47 HPV-positive cases, 4(2%) contained HPV-16 and 11(6%) contain HPV-18 genotypes.^19^ This do not co-relate with our findings, as we found HPV-16 in 03/35 controls and HPV-18 DNA was not detected in any of the salivary samples of normal controls.

In a study from Karachi, Pakistan, on the molecular analysis of HPV detection in tissue blocks by Dot Blot and in-situ hybridization and PCR method found 17.7% cases of OSCC positive for HPV-16 & 18 DNA on PCR and 14.6% cases showed viral DNA by NISH. While in only 4.6% cases of oral PML, the viral DNA was detected. They concluded that high risk HPV-16 & 18 has contributing co-factor role rather than mandatory causative role in oral carcinogensis.^14^

A study from Allahabad, India, reported 27.4% HPV detection in OSF while it was detected in 31.53% cases of OSCC on brush biopsy specimen by Hybrid Capture II test. PCR was done on the same specimen for the detection of E6 DNA of HPV-16. They found detection rate of 25.96 in OSF cases and 32.43% in cases of OSCC.^20^ Same group reported high risk HPV detection rate of 31.42%(33/105) in oral PML (OSF) by Hybrid capture II test.^21^ Our findings regarding detection of HPV DNA in OSCC cases is quite close to the findings of Allahabad study but the findings in oral PML (OSF) does not co-relate. Another study from Karnataka, India found salivary detection rate of 54.2% for HPV-18 in OSCC cases. They reported multiple mixed HPV infection in 4.18% cases of OSCC in India.^22^ These finding are in accord to our study.

In a meta-analysis (1988-2007), the incidence of HPV in head & neck SCC versus OSCC, it was observed that pooled prevalence of HPV in the tissue samples of head and neck SCC was 34.5% while in cases of OSCC, it was 38.1%.^23^ These findings are in close proximity to our study.

Significant association was reported in OSCC with HPV-16 and to a lesser extent with HPV-18. In the salivary samples HPV-16was the most detectable virus reported in the literature.^10^,^13^,^14^,^22^-^24^ Low copy numbers of HPV was found in the oral PML in the previous studies.^25^,^26^ These findings are in conjunction with our study.

It is difficult to compare various published data on HPV association as there are wide differences in findings. Possible reasons of this disparity is variation in parameters studied such as type of samples (biopsy tissue, scraping, oral rinse or saliva) preparation method (fresh, frozen or fixed), type of molecular assay employed for DNA extraction, sensitivity of the methodology employed, status of the disease, ethnic and geographical differences in genetic, environment, social, cultural and sexual habits of the population studied.^27^,^28^

Comparing various studies, the results are highly variable and controversial. No clear cut association or relation of HPV infection with oral PML and OSCC has so far emerged. Our findings are consistent with many of the reported studies in literature. We are unable to support definitive causative or mandatory initiator role of HPV in oral carcinogenesis in our etiologically distinct population where use of areca nut and chewable tobacco is very prevalent in the society.

## CONCLUSION

Host response towards HPV infection for the causation of oral cancer cannot be fully established. More over different genetic makeup, environmental and geographic differences, indulgence in peculiar risk factor habits, different sexual practices compared to west and difficult clinical evaluation of acquisition of viral load due to socio-cultural and religious restrictions could be the reasons. Our study is unable to support definite causative or mandatory initiator role of HPV infections in oral carcinogenesis, it may have a co-factor role rather than just a passenger virus.
